# Studies of life history of *Gagea graeca* (*Liliaceae*) based on morphological and molecular methods

**DOI:** 10.1186/s40529-017-0194-6

**Published:** 2017-10-03

**Authors:** Martin Schnittler, Akmaral Nursafina, Angela Peterson, Jens Peterson, Carl Barnick, Anja Klahr

**Affiliations:** 1 Institute of Botany and Landscape Ecology, Ernst Moritz Arndt University Greifswald, Soldmannstr. 15, 17487 Greifswald, Germany; 2 Institute of Biology, Martin-Luther-University of Halle-Wittenberg, Weinbergweg 10, 06120 Halle/Saale, Germany; 3 State Office for Environmental Protection of Saxony-Anhalt, Reideburger Str. 47, 06116 Halle/Saale, Germany; 4 L.N. Gumilyov Eurasian National University, 5 Munaytpassov Str, 010008 Astana, Kazakhstan

**Keywords:** Amplified fragment length polymorphism (ALFP), Drought adaptation, Reproductive biology, Resource allocation, Seed set

## Abstract

**Background:**

We studied the life history of *Gagea graeca* (L.) A. Terracc. (sect. *Anthericoides*) by field surveys on the Greek island of Crete, including quantitative analyses of 405 individuals, estimation of resource allocation by measuring the nitrogen content of different plant organs, assessing seed set and recording genetic diversity via amplified fragment length polymorphism (AFLP) analyses. In contrast to most species of the genus *G. graeca* seems to be a short-lived perennial, developing several characters that are rather typical for annual plants.

**Results:**

Although seed set varies largely, flowering plants produce many (68 ± 79) small, flattened seeds (mean weight 73 ± 22 µg) in comparison to a single bulbil. If measured as nitrogen content of the respective plant parts, investment in seeds (25%) is much higher than that in bulbils (4%). In addition, the threshold for flower formation (expressed as bulb size where 50% of the plants form the respective structure) is with 2.17 ± 0.05 mm lower than that for bulbils with 2.80 ± 0.16 mm. This is in accordance with AFLP analyses revealing predominantly sexual reproduction (only 9.1% of 110 investigated plants belonged to clones).

**Conclusion:**

In the genus *Gagea* early, predominantly sexual reproduction seems to be characteristic for species from arid habitats, coupled with a low proportion of clonal plants.

## Background

*Gagea graeca* (L.) A. Terracc. is one of the few white-flowering species in the genus *Gagea* Salisb. (*Liliaceae*). Together with the closely related *G. trinervia* (Viv.) Greuter (Peruzzi et al. 2008) it is assigned to section *Anthericoides* A. Terracc. This monophyletic section (e.g., Peruzzi et al. 2008; Peterson et al. 2008, 2011, 2016; Zarrei et al. 2009) within the genus *Gagea* (incl*. Lloydia* Rchb.; see Peterson et al. 2004, 2008; Peruzzi et al. 2008; Zarrei et al. 2011) is currently accepted in all infra-generic classifications (Levichev in Peterson et al. 2008; Zarrei et al. 2011; Peruzzi 2012a). Both species are endemic to the Mediterranean region and are diploid (2*n* = 24; Peruzzi 2003, 2008, 2012a). In several phylogenetic studies (e.g., Peruzzi et al. 2008; Peterson et al. 2011) the section *Anthericoides* was found to be in a sister position to other studied sections of the genus.

Plants of *G. graeca* germinate with a single leaf, but in subsequent years the number of basal leaves grows to 2, (sometimes to 3 or 4; Levichev in Peterson et al. 2008). According to Peruzzi et al. (2008) plants can produce a first flower already in their second year. In adult plants usually several flowers were observed. Compared with the size of the plant, flowers are showy, with white, elongated tepals with reddish veins, which are recurved at apex. This habit, resembling that of an annual plant, is unusual among the genus and points to prevailing reproduction via seeds, whereas most other species of the genus reproduce predominantly via bulbils (e.g., Levichev 1999, 2013; Schnittler et al. 2013). However, in addition to the renewal bulb produced in the axil of the first leaf (obligatory for the genus, furthermore named parent bulb) plants with two leaves start to produce a first bulbil on a very short stolon (hypopodium according to Peruzzi et al. 2008). As such, vegetative reproduction is regularly available as a second option. The other species of section *Anthericoides*, *Gagea trinervia* differs from *G. graeca* in “having bulbils with stolon-like hypopodium, a regular sister bulbil at the immature stage, an unifacial, juvenile-like basal leaf to adult stage, few cauline leaves, few flowers and a low level of sexual reproduction” (Peruzzi et al. 2008).

**Fig. 1:**
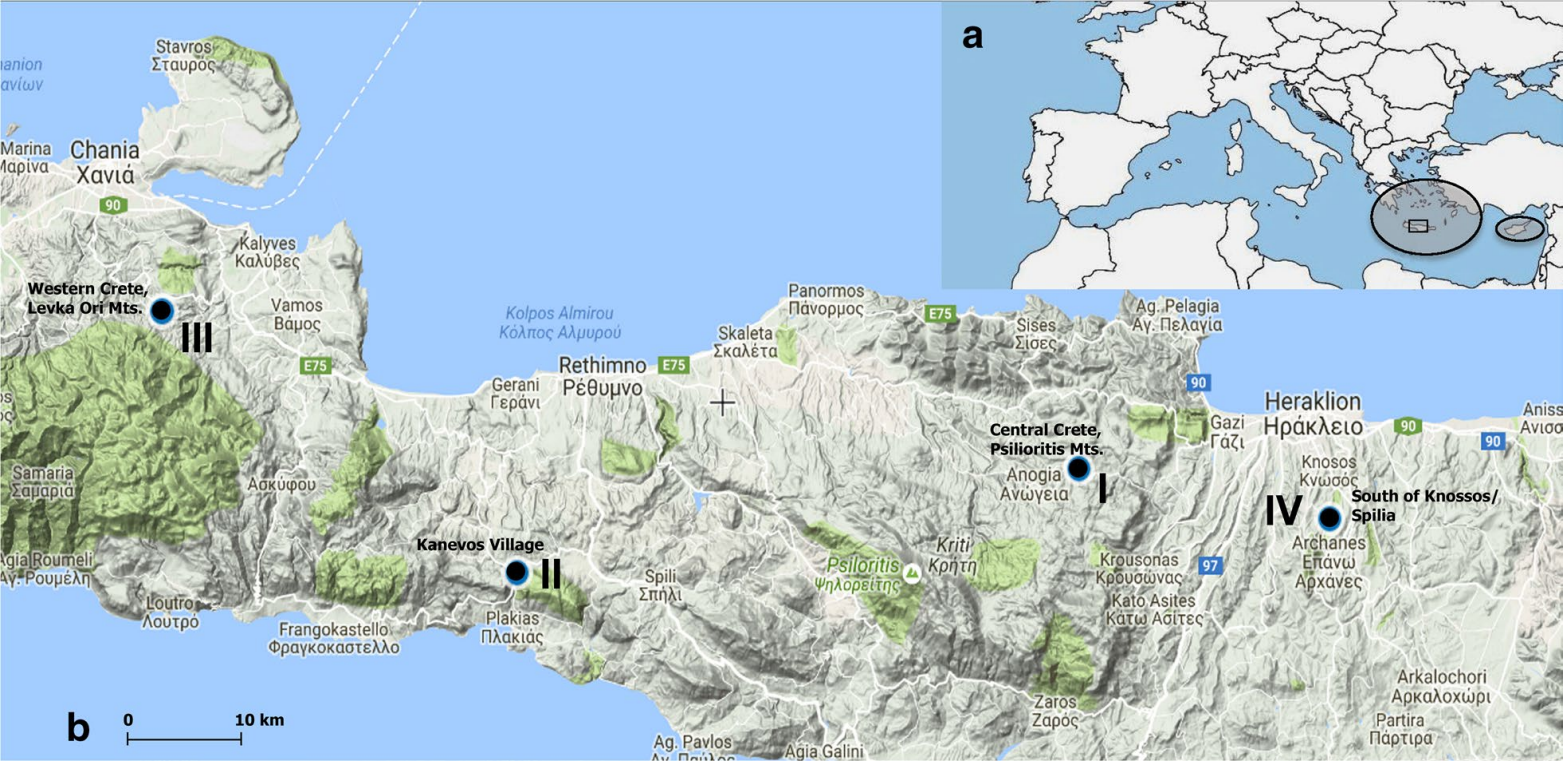
**a** Distribution of *Gagea graeca* in the Mediterranean region (encircled regions).** b** Collecting sites for the four studied populations from the central part of the island of Crete (marked with an rectangle in **a**); map compiled using the geospatial conservation assessment tool GeoCAT (http://geocat.kew.org/)

*Gagea graeca* occurs in the eastern Mediterranean region, with an apparent center of occurrence in Greece and the Aegean islands (Fig. 1a; Jahn et al. 1995; Strid 2016). (Tison 2016) provide a preliminary map of the distribution of *G. graeca* together with a threat assessment of based on the IUCN categories. According to these authors, the species is found in southeastern Sterea Ellas and extends north to the southern Thessalian seashores, in most of the Peloponnese and many Aegean islands, including Crete and Rhodos (but seems to be absent from some northeastern islands and the Karpathos group). It is part of the flora of Turkey (Tekşen and Erkul 2015) and reported from southwestern Anatolia (provinces of Izmir, Antalya and Muğla) and Cyprus. Records from Israel are likely to be erroneous (the species is not mentioned in Danin and Danin 2015). On the contrary, *G. trinervia* occupies a small and highly disjunct range, occurring in on the Italian Island of Sicily and in the northeastern part of Libya (Peruzzi et al. 2008).

*Gagea graeca* can be found in various, usually slightly disturbed, open habitats like dry grasslands, rocky slopes and fallow fields especially on soils that are provided with some moisture to prevent the relatively small bulb from desiccation over the summer. In Crete, where the species was investigated, most of the ancient woodlands are now replaced by low, thorny shrubs (a vegetation called “phrygana”) and open grasslands (Zimowski et al. 2014). Here *G. graeca* usually inhabits small gullies in where the vegetation cover does not exceed 50%. At such places, the plant can form large populations comprising thousands of individuals.

This investigation was undertaken to study adaptations of *Gagea graeca* on its Mediterranean environment, and compare these adaptations with those of another species of the genus adapted to arid environments, *G. bulbifera* (Pall). Salisb. (Beisenova et al. 2015). A quantitative morphology approach (Schnittler et al. 2009, 2013; Beisenova et al. 2015), including measurements on living plants of all ontogenetic stages, is used to quantify possible traits enabling *G. graeca* to persist in the only temporarily wet vegetation Mediterranean shrub land. In addition, amplified fragment length polymorphism (AFLP), (Vos et al. 1995) was employed to estimate the proportions of vegetative (leading to clones) and generative reproduction (producing new genotypes) in the species.

## Methods

### Study region

**Fig. 2:**
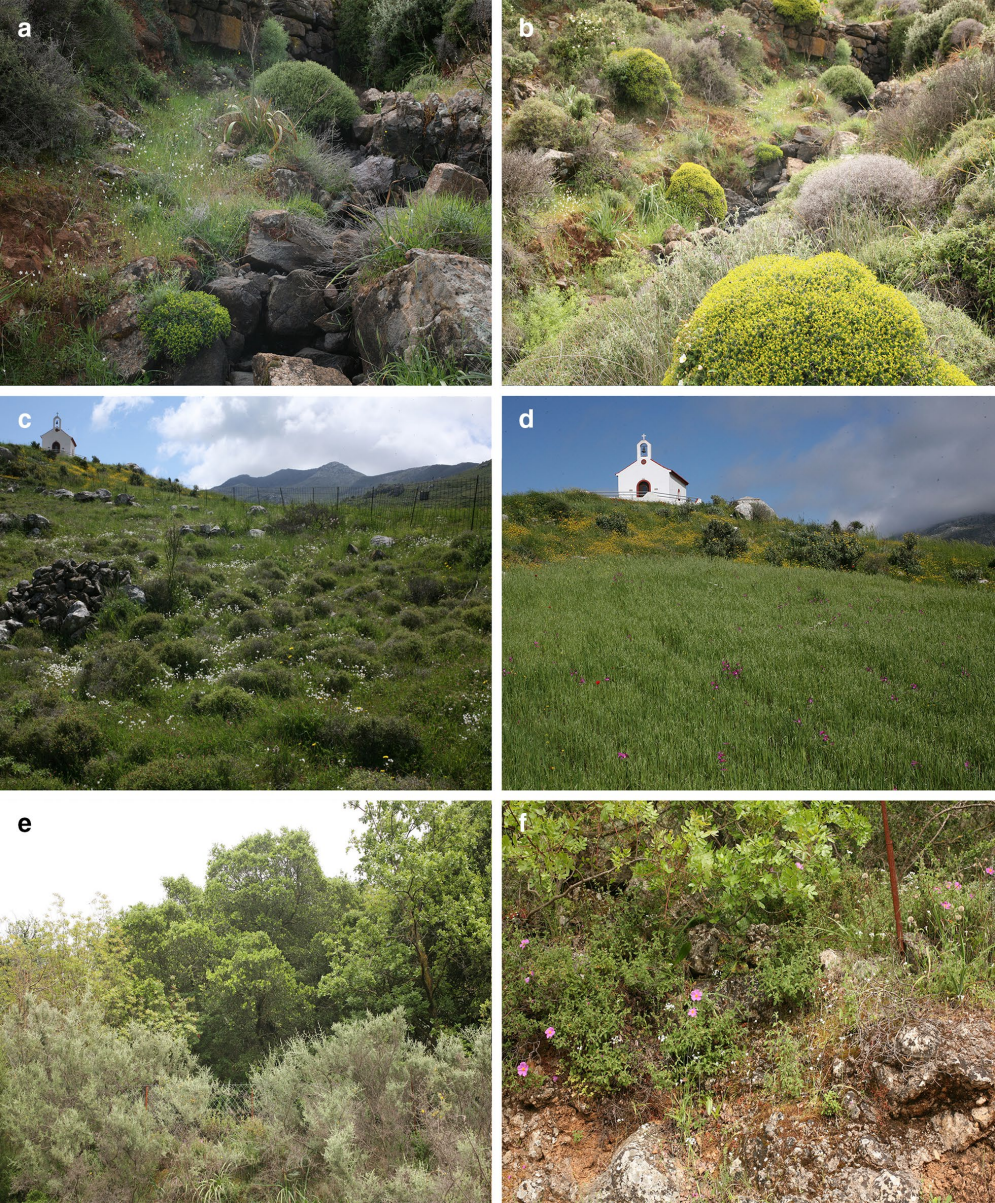
Growth sites for *Gagea graeca*. **a**, **b** Population I along a small gully in the phrygana of the Psilioritis Mts. **c**, d Population II at a temporarily wet fallow field. **e**, **f** Population III at former olive orchards, now invaded by holm oak, at a slope

*Gagea graeca* was investigated during spring of 2013 and 2014 at the island of Crete, where the species is common (Jahn et al. 1995). In these habitats that are often disturbed by grazing livestock, *G. graeca* inhabits slightly disturbed places especially where at least some soil moisture will be preserved during the summer, to prevent the bulbs from desiccation (Fig. 2). Table 1 gives numbers of plants investigated for morphological measurements, C/N data, seed set and AFLP analyses.

**Table 1:** Numbers of plants investigated for morphological measurements, C/N data, seed production and seed weight, and AFLP analyses from the four investigated populations

Population	I	II	III	IV	Total
Morphology	184	221	–	–	405
Seed production	59	64	–	54	177
Seed weight	8	10	–	6	24
C/N data	14	–	16	12	42
AFLP analyses	54	56	–	–	110

Four populations of Crete (Fig. 1) were investigated (numbered I to IV; Fig. 1b, Table 1):

I. Central Crete, Psilioritis Mts., Anogia, along a little brook in phrygana ca. 1 km SE Gonies, road fom Gonies to Anogia, at a branch that leads to a windmill, slopes with fine soil between rocks (flysch), elev. 600 ± 25 m a.s.l., 35°17′58 “N, 24°55′36″ E ± 25 m,

II. Central Crete, southern coast near Plakias, temporarily wet fallow field ca. 800 m SE Kanevos village, along the gravel road to a small chapel, ascent to Kouroupu hills, elev. 476 ± 25 m a.s.l., 35°13 ‘37″ N, 24°24′10″ E ± 25 m,

III. Western Crete, Levka Ori Mts., Chania, rocks and fallow Olive orchards in *Quercus ilex*/*Pistacia lentiscus* woodlands: about 1 km N Tsakistra village, road between Tsakistra and Kampoi, elev. 581 ± 30 m a.s.l., 35°24′33″ N, 24°04′16″ E ± 25 m,

IV. Central Crete, south of Knossos/Spilia near Iraklio, phrygana in succession to *Quercus* shrubland: ca. 1450 m SW of Kera Eleousa village, elev. 222 ± 20 m a.s.l., 35°15′52″ N, 25°09′38″ E ± 25 m.

### Morphological investigations

All morphological studies were carried out on living plants towards the end of the flowering season (March 15–30, 2014) from populations I and II (Table 1). At this time, most of the plants already developed fruits (capsules); a few plants were still in bloom. Of these 405 plants, 114 were in non-flowering condition.

Using a ruler and/or a digital caliper (precision ± 0.02 mm) we measured the diameter of the replacement bulbs (including the thin tunic of the former parent bulb; see Schnittler et al. 2013) and the diameter/length of the usually single basal bulbil and its short to extremely short stolon. In addition, we recorded number of flowers, shoot height above ground, and numbers of basal and stem leaves. For all measures, the standard deviation (SD) is given. To determine a threshold for the diameter of the replacement bulb needed to develop bulbils and/or flowers, these graphs were fitted against the equation y = 1/[1 + e^−(x−xo)/b^] which describes a sigmoid function with xo as the threshold (the turning point of its slope). For the graphs, flowers with only the male function (aborted and much smaller capsules) were counted as 0.5, perfect flowers as 1 (capsules are of normal size and fertile).

### Seed production

About a month later (between May 1 and May 20), the number of seeds per plant was counted for 177 plants from populations I, II and IV (Table 1). To record variation in seed production, all formerly flowering plants (those with a developed shoot) for a certain area (1–2 m^2^) were collected. At this time, capsules were fully developed and all vegetative plant parts were dried up. In a few plants capsules started to dehisce; such plants were not collected.

### Resource allocation

Material from populations I, II and IV (Table 1) was used to determine absolute contents of carbon and nitrogen. Plants were dried for about 4 days in phosphorus pentoxide, and dry mass of the respective plant parts (seeds, replacement bulbs and vegetative structures) was determined with a precision of ± 1 µg (for seeds, 100 seeds were pooled for weighing). Several items of these plant structures were cut or pooled in a way that dry mass ranges between 6 and 15 mg. This weight corresponds with one average-sized replacement bulb, vegetative remains from a single plant, or ca. 100 seeds. Using replicate samples, a total of five replicates were measured except for basal bulbils. This procedure yielded 42 samples in total which were wrapped into tin foil and analyzed for carbon (C) and nitrogen (N) content with an automatic analyzer Vario EL III. Using the means of 3–10 samples, N-contents were calculated and used as a resource estimator (compare Ashman 1994). Figures for N content per mg dry mass were used to estimate nitrogen content of the respective plant parts, and calculate their relative investments in terms of nitrogen incorporated.

### AFLP analyses

For a total of 110 sampled plants from populations I and II (Table 1) AFLP profiles were generated, using the methods described in Pfeiffer et al. (2011). For these analyses two transects (populations I and II with 27 and 28 pairs of plants, respectively, see Table 1) were sampled. We employed the sampling scheme of Pfeiffer et al. (2011), where pairs of plants (maximum distance 10 cm) were collected along a transect line (minimum distance between pairs 1 m).

We did five/six full replicates (including DNA extraction) for the first/second transect to determine the error rate. Due to the high DNA content of many *Liliaceae* (Veselý et al. 2012; Zonneveld et al. 2015), we used a primer combination (EcoRI-AGCG plus Vsp-GCAT) with four selective bases. The first selective base was added to the pre-amplification primers, and all four were added to the primers used in the main amplification. The electroferograms obtained with a sequencer ABI 310 (applied biosystems) were binned and the alleles, defined as peak presence/absence, were read automatically to a 0/1 matrix using GenMapper 4.0 software (applied biosystems). To identify clones, an algorithm was programmed in Microsoft Excel which does not only check for identity, but allows to define a threshold to account for genotyping (biological, experimental, and scoring) errors (Douhovnikoff and Dodd 2003; Schnittler and Eusemann 2010). This threshold was derived from histograms of pairwise distances between samples. The histograms showed a bimodal distribution with two peaks, one for non-identical plants, and one for putative clones, with a local minimum (the threshold) in between. This threshold was below the pairwise distances between replicates. All samples with AFLP profiles deviating from each other in more alleles than defined by the threshold were regarded as independent genotypes.

## Results

### Morphology

**Fig. 3:**
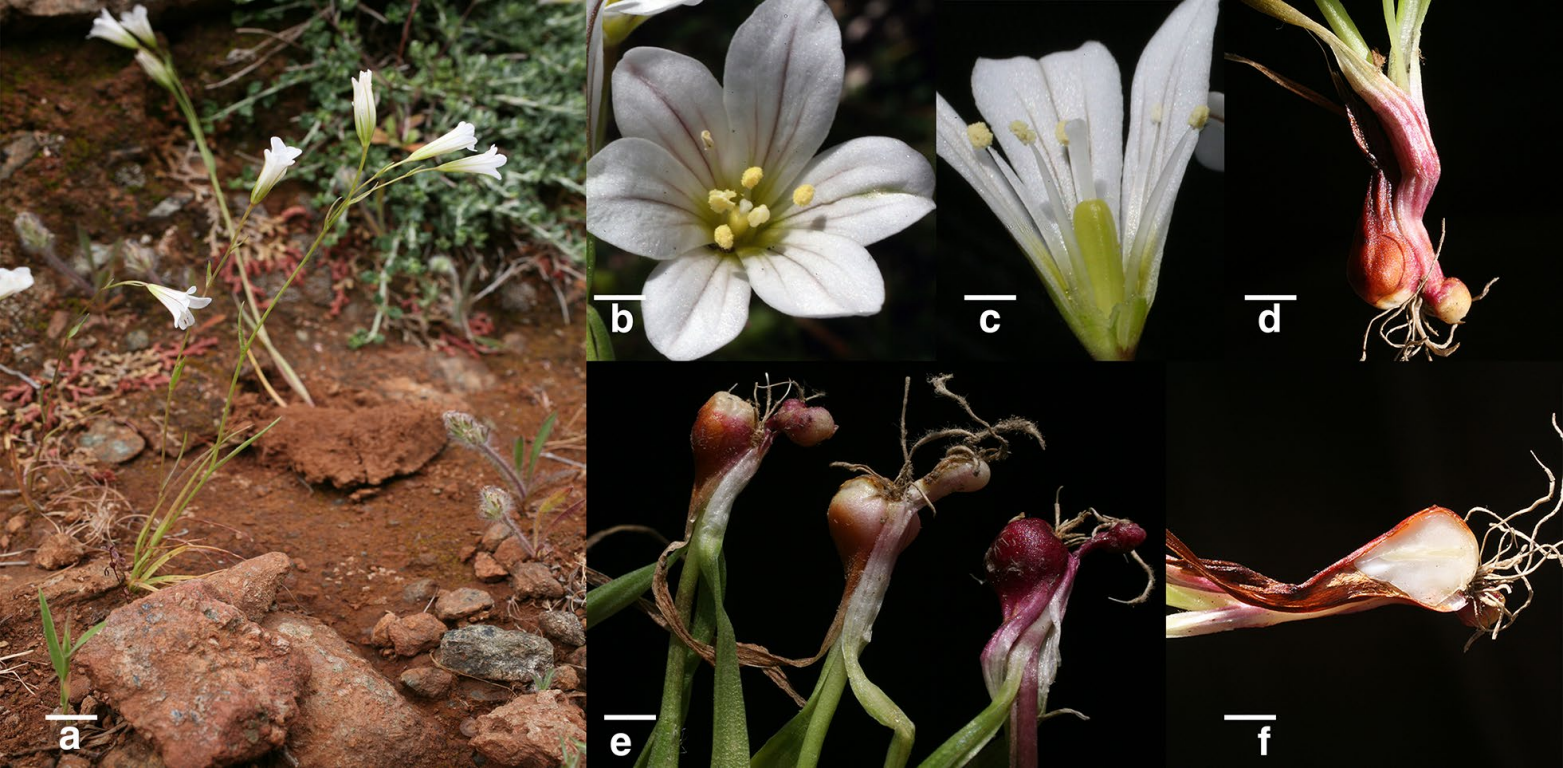
Morphology of *Gagea graeca*. **a** Strong flowering plant with two scapes and four basal leaves. **b** Closeup of a flower showing reddish veins (the color is even more pronounced at the outer side). **c** Longitudinal section through a flower, showing the blunt capsule. **d** Basal part of a flowering plant with one bulbil, showing the reddish tunics formed by the sheaths of basal leaves. **e** Three flowering plants showing variation in stolon length; the plant on the right comes close to the maximum. **f** Longitudinal section through a replacement bulb. Bar length indicates in **a** 1 cm, **b**–**e** 3 mm, **f** 2.5 mm

Although *Gagea graeca* was always seen in populations of several hundred plants or more, the slender growth makes the plants quite inconspicuous. Only at flowering time, the plants are rather conspicuous (Fig. 3a), developing 1–3, rarely more, white flowers.

All measurements of morphological characters are summarized in Table 2. Large non-flowering plants develop two, more rarely three basal leaves. In flowering plants, which may have up to four basal leaves of 3–8 cm length, an upright shoot (82 ± 60 mm tall, range 25–414 mm) develops. In large flowering plants, the axils of the basal leaves can develop additional shorter shoots with usually one flower. The 2–6 scape leaves are much shorter and rarely exceed 4 cm in length, in the axils of the upper in addition to the single terminal flower further flowers may develop. About one-third of all plants (98 of 291 flowering plants) developed only a single terminal flower; larger plants carry additional lateral flowers, but these are often much smaller and seem to be predominantly male, since ovaries are often underdeveloped. On average, flowering plants developed 1.9 ± 1.1 (1–7) flowers. The white tepals are obovate (Fig. 3b, c), broadest above the middle and show one larger central and two smaller lateral green veins, which turn conspicuously reddish towards the tip of the tepal. The six anthers are very pale yellow. Developing flowers are nodding and become upright orientated only during anthesis. Only a part of the flowers (mostly the terminal ones) develop into strictly upright capsules which can reach up to 12 mm length; the style remains usually on the tip of the capsule.

**Table 2:** Life history characteristics of two species of *Gagea* from arid environments (*Gagea graeca*, *G. bulbifera*) compared with a species from temperate deciduous woodlands (*G. lutea*)

Species	*G. graeca*	*n*	*G. bulbifera*	*n*	*G. lutea*	*n*
Reference	This study		Beisenova et al. (2015)		Schnittler et al. (2009), Pfeiffer et al. (2011)	
*Morphology*						
Bulb size, flowering plants	3.01 ± 0.63	*291*	2.55 ± 0.48	*291*	8.94 ± 1.62	*239*
Threshold (mm bulb diam.) for						
Flowers	2.17 ± 0.05	*405*	1.90 ± 0.13	*417*	6.94 ± 0.04	*505*
Bulbils	2.80 ± 0.16	*405*	4.96 ± 11.39	*417*	2.63 ± 0.21	*505*
Difference	−0.63	*405*	−3.06	*417*	4.31	*505*
Bulbils per plant showing bulbils (range)	1.01 ± 0.10 (1–2)	*194*	1.00 ± 0.00	*26*	8.65 ± 5.31 (1–23)	*226*
Average bulbil size	1.26 ± 0.39	*194*	1.11 ± 0.48	*26*	1.49 ± 0.42	*226*
Switch present?	No	*405*	No	*417*	Yes	*505*
*Seed set*						
Flowers per plant	1.9 ± 1.1 (1–7)	*177*	1.4 ± 0.9 (1–9)	*230*	3.2 ± 1.5 (1–8)	*217*
Capsules per fruiting plant	1.2 ± 0.6 (1–4)	*123*	1.4 ± 0.9 (0–9)	*210*	3.2 ± 1.5 (0–8)	*216*
Mean seed weight (µg)	73 ± 22	*24*	80 ± 20	*10*	1650 ± 340	*10*
Seeds per flowering plant	68 ± 79 (0–445)	*177*	119 ± 90 (0–827)	*230*	41 ± 30 (0–159)	*217*
Seeds per fruiting plant	98 ± 77 (0–445)	*123*	130 ± 86 (0–827)	*210*	41 ± 30 (0–159)	*216*
Seeds per capsule	80 ± 39 (0–184)	*123*	99 ± 40 (0–224)	*210*	12 ± 6 (0–33)	*216*
*Resource allocation*						
N-content (µg/mg dry weight)						
Replacement bulbs	27.9 ± 2.6	*9*	23.9 ± 1.8	*5*	8.3 ± 3.5	*10*
Seeds	41.2 ± 6.6	*24*	22.1 ± 1.8	*5*	25.7 ± 1.9	*10*
N investment (%) into						
Replacement bulbs	67.1	*9*	36.4	*5*	25	*10*
Vegetative structures	3.8	*9*	21.1	*5*	ca. 60	*10*
Basal bulbils	3.9	–	0.5	*1*	–	–
Axillary bulbils	–	–	4.5	*5*	–	–
Seeds	25.2	*24*	36.4	*5*	> 15	*10*
*AFLP data*						
Diversity R = (G−1)/(N−1)	0.95	*110*	1.00	*10*	0.56	*141*
Proportion of clonal plants	0.09	*110*	0.00	*10*	0.62	*141*

Mean values ± SD and ranges (in parentheses) are given as well as the number of plants or structures (n) investigated. For seed weight, 100 seeds were counted and weighed together. Due to the switch in *G. lutea* (flowering plants do not produce bulbils any more), investment in bulbils and flowers cannot be compared directly for this species. Data sets for morphology, seed set, resource allocation and AFLP analyses include different plants

The replacement bulbs are always close (1–3 cm) to the surface, and are hidden by the reddish-brown sheaths of the basal leaves. The first basal leaf, which is as well the first to decay during flowering season, develops the replacement bulb; the second, very rarely the third as well, develops usually one basal bulbil which is on a saccate enlargement of the basal leaf, which forms a short stolon. Larger non-flowering plants (34 of 114, mean bulb diam. 2.20 ± 0.40) developed a bulbil (Fig. 3d–f). From 291 flowering plants measured, the weaker individuals (131 plants, mean bulb diam. 2.72 ± 0.56) did not yet develop a bulbil, the larger ones (160 plants, mean bulb diam. 3.24 ± 0.69 mm) supported a basal bulbil (diam. 1.26 ± 0.39 mm). Among these were two with a second, smaller basal bulbil (sister bulbil; diam. 1.17 ± 0.36 mm). Bulbils developed often on a short (0.42 ± 0.71 mm, range 0–4.01 mm) stolon. Compared with other species in the genus, the replacement bulbs of *G. graeca* stay small even in richly flowering plants; the maximum bulb size recorded by us was 5.12 mm.

**Fig. 4:**
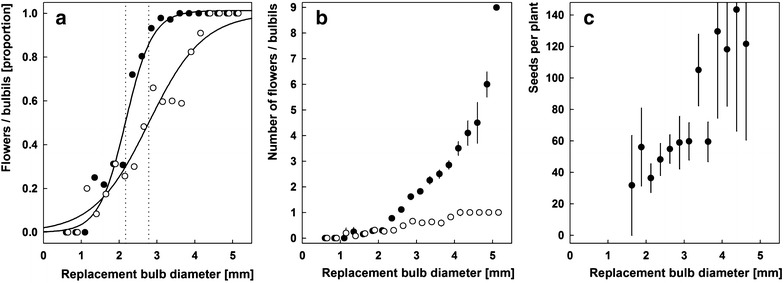
Reproductive pattern of *Gagea graeca*, shown are cohorts of plants corresponding to classes of 0.25 mm diameter width of the replacement bulb (n = 405). **a** Proportions of plants forming flowers (black circles) and a basal bulbil (white circles, the latter are printed with a slight shift to the right to make overlaps with the black circles visible). Graphs were fitted against the equation y = 1/[1 + e^−(x−xo)/b^] with xo as the threshold for the formation of the respective structure (indicated by a vertical dotted line). **b** Average numbers of basal bulbils (white circles) and flowers (black) per plant in the respective diameter classes. **c** Seed set. Bars for the latter two plots denote the standard error of means

Figure 4a, b shows flower and bulbil formation in dependency from the size of the replacement bulb. Only strong plants (exceeding 4 mm bulb diameter) develop always a bulbil. The threshold for flower formation is lower (2.17 ± 0.05 mm bulb diameter) than the threshold for the formation of the basal bulbil (2.80 ± 0.16 mm).

### Seed production

A total of 177 plants from three populations (Table 1) with altogether 339 flowers and 165 seed capsules were counted. Seed production increased strongly with bulbil size class (Fig. 4c). Plants had on average 1.9 ± 1.1 (1–7) flowers, but the single flower of weak plants and the terminal flower(s) of larger plants did often fail to set seeds. Therefore, only 123 of 177 investigated plants were fertile; this cohort developed 1.2 ± 0.6 capsules with 98 ± 77 (0–445) seeds.

**Fig. 5:**
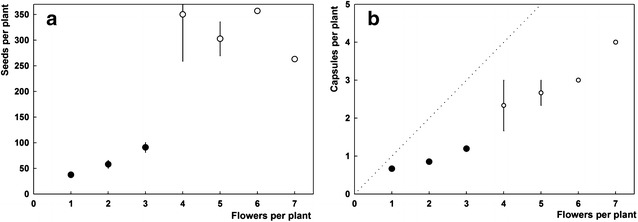
Seed set for *Gagea graeca* in dependence form the numbers of flowers (**a**); and the relation of capsules per flower (**b**). Most plants hat 1–3 flowers (these classes are represented by at least 40 plants each, black dots), whereas only eight plants had 4–7 flowers (white dots). Bars indicate the standard error of means

Apart from the 54 sterile plants, most individuals developed one capsule (n = 124, 69 ± 45 seeds), eight plants two capsules (112 ± 97 seeds), seven plants three capsules (333 ± 97 seeds), and one plant four capsules (263 seeds). Seed set in plants increased not proportionally with the number of flowers (Fig. 5a). Only about two-thirds, in plants with more flowers even less, of the flowers developed seed capsules (Fig. 5b). Seeds are very lightweight: average seed weight (measured for samples of 100 units) was 86 ± 22, 75 ± 14 and 48 ± 5 µg for populations I, II and IV (10, 8, and 6 samples are 100 seeds measured, respectively), and population IV differs significantly from the two other populations (Mann–Whitney U test, p = 0.05).

### Resource allocation

**Fig. 6:**
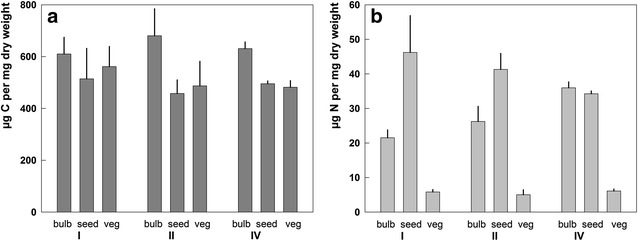
Investment of carbon (**a**) and nitrogen (**b**) in different plant parts for three populations (I, II and IV) of *Gagea graeca*. Shown are mean ± SD of elementary contents (µg per mg dry weight) for bulbs (bulb, n = 3), seeds (seed, n = 6–10) and vegetative plant structures (veg, n = 3)

Table 3 shows carbon and nitrogen content in different plant structures. For the three investigated plant structures replacement bulbs, seeds and vegetative parts C contents are comparable (Fig. 6a), whereas N content is much higher in seeds and bulbs (Fig. 6b). Except for population IV, where seeds had a much lower average weight, seeds store 1–1.5 times more N per unit dry mass than bulbs.

**Table 3:** Mean N and C contents measured for bulbs, seeds and vegetative parts of *Gagea graeca*

Plant organ	*n*	N (µg/mg)	C (µg/mg)	C/N
Bulbs	*9*	27.9 ± 2.6	641.5 ± 62.5	22.9
Seeds	*24*	41.2 ± 6.6	486.0 ± 72.6	11.8
Veg. structures	*9*	5.6 ± 0.8	510.1 ± 62.6	90.3

Samples were pooled to reach a total weight between 6 and 15 mg (equaling one bulb, 100 seeds, and vegetative parts of 3 plants)

With known amounts of plant structures used for measurements, the relative investment of a plant into the respective structures can be estimated. The N content of an average-sized replacement bulb was measured as 470.40 ± 20.05 µg (n = 9). Bulbils were too small and occurred too rarely to measure N content. If the size of a bulb is approximated by the volume of a sphere with the diameter of the respective structure (replacement bulbs: 3.24 ± 0.69 mm; basal bulbils 1.26 ± 0.39 mm), the ratio between average volumes of replacement bulbs and bulbils is 17:1. If we assume that bulbils have the same N-content per unit volume as replacement bulbs, the N content of an average bulbil can be estimated as 27.67 µg. The absolute N content for 100 seeds was measured as 259.97 ± 66.53 µg. Since a plant forms on average 68 ± 79 seeds (including plants with lacking seed set, see Table 2), the N content of 68 seeds would be 176.78 µg. Similarly, the vegetative parts of nine plants were measured (N content 79.10 ± 4.56 µg). Comparison of the absolute N contents of these structures gives the following picture: flowering plants of *G. graeca* allocate most of their resources (67.1%) to the replacement bulbs, 3.9% to the usually single basal bulbil, 25.2% to an average of 68 seeds, and 3.8% remain in vegetative plant structures.

### Genetic diversity

**Fig. 7:**
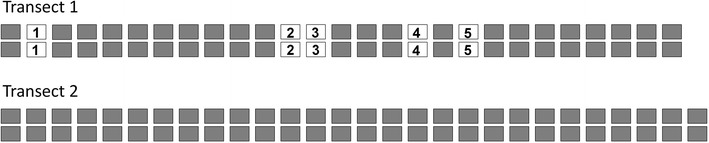
Genetic diversity of two transects of *Gagea graeca* (populations I and II) comprising 27 and 28 pairs of plants, respectively. Grey rectangles without numbers represent plants with unique genotypes; white rectangles indicate plants belonging to clones which are numbered

The AFLP analysis with four selective bases resulted in a total of 299 (population I) and 259 (population II) readable alleles (peaks taller than 150 relative fluorescence units). With fully automated bin set and scoring we obtained on average 27.0 ± 4.5 (range 22–33, population I) and 25.9 ± 12.4 (range 10–43, population II) deviating peaks between replicates for populations, translating to error rates of 10.4 and 8.3%, respectively. Although this figures are rather high, the histogram for the pairwise comparisons of all samples (classes of five differing alleles) showed a clear gap between allele differences for replicates/clonal samples and all others, leading to upper thresholds for clonal identity of 40 and 28 deviating alleles, respectively. The non-clonal samples differed on average in 75.1 ± 8.1 (range 43–98) and 61.7 ± 10.9 (range 29–103) alleles (populations I and II, respectively). Population I revealed five pairs of plants that were assigned to clones (Fig. 7), population II consisted of singletons only. This correlated with a lower genetic diversity for the first population, if expressed as the mean Jaccard distance (I: 0.48 ± 0.04; II: 0.57 ± 0.08). In total, 9.1% of all investigated individuals belonged to clones.

## Discussion

*Gagea graeca* differs from other species of *Gagea* in the Mediterranean area (which are mostly members of the section *Didymobulbos*, Tison et al. 2013) by its slender habit and the solitary growth of plants. Typically, in species of *Gagea* relying on reproduction by subterranean bulbils the plants form clusters, whereas plants of *G. graeca* usually grow apart from each other. A high proportion of the plants in all populations collected by us were always seen in flowering condition. Therefore, sampling for the morphological measurements focused on small, non-flowering plants, to have all classes of parent bulbil size more evenly represented than it would be the case in a random sample from the population. We nevertheless could only achieve a proportion of 28% non-flowering plants. Although *G. graeca* is certainly a short-lived perennial indicated by the significant resource allocation to replacement bulbs (and true annuals are not known for *Liliaceae*, Peruzzi 2016) the habit of the plant with its comparatively large flowers resembles that of an annual. In addition, in agreement with Greuter (1970) in our quantitative study we rarely observed sister bulbils in *G. graeca* (two of 194 plants were seen with two bulbils). Peruzzi et al. (2008) did not found such a structure at any stage of the ontogeny (herbarium collections, cultivated plants).

Measuring or counting a number of morphological traits as demonstrated in Schnittler et al. (2009) quantifies these visible adaptations and allows to relate them to the resource status of the plant, here estimated by the size of the replacement bulb, which carries the plant through the summer dormancy period into the next year (Levichev 1999, 2013). These measurements confirm reports about plant ontogenesis (Peruzzi et al. 2008). Although the age of the plants cannot be determined, the measurements allow to determine threshold (in terms of bulbil size) for the development of seeds and bulbils. For the species of the genus *Gagea*, the relation between sexual (seeds) and asexual (bulbils) reproduction seems to be a crucial adaptive trait (Schnittler et al. 2013) and is determined by these thresholds (as the diameter of the replacement bulb where 50% of all plants develop the respective structure, Schnittler et al. 2009) and the number of seeds and bulbils. Therefore, resource allocation into seeds and bulbils can be assumed to be under high selective pressure. Several traits should influence the seed/bulbil ratio. First, seeds show a higher desiccation tolerance than bulbils, making them true dormant stages. A second factor, often seen in the genus *Gagea*, is partial or full sterility caused by high (and especially odd) ploidy levels. This constrains a higher investment into bulbils as the only remaining mode of reproduction. Third, large genome size, as often found in monocotyledonous plants, especially *Liliales*, (Leitch et al. 2010) tends to complicate meiosis and thus sexual reproduction. Taking these factors into account, species of *Gagea* in arid habitats should be diploid (or add least have a low even ploidy level) and have small genomes to allow predominant reproduction via seeds as the more drought resistant diaspore type. Indeed, a meta analysis of *Liliaceae* indicated counter selection against large genomes in arid climates (Carta and Peruzzi 2016).

If the environment allows, increased bulbil production can function as an exit strategy for missing seed set, as shown by a comparison between *G. lutea* (hexaploid) and *G. spathacea* (nonaploid, Schnittler et al. 2009): the latter species compensates missing seed set, most likely caused by incorrect pairing of chromosomes due to their odd number, with an increase in the number of basal bulbils and seems to be fully clonal (Pfeiffer et al. 2011). To compare the amount of resources invested into both types of diaspores, we used nitrogen content as a proxy (compare Ashman 1994). Shifts in the bulbil/seed ratio have a strong impact on clonal diversity of a species (since not only numbers, but also establishment probabilities of the respective diaspore types matter), but are as well recognizable by different resource allocation and changes in numbers of these propagules.

Table 2 shows a comparison of the life history traits of a species from humid environments (*G. lutea*, Schnittler et al. 2009) and two species from arid environments, (*Gagea graeca*, this study, *G. bulbifera*, Beisenova et al. 2015). *Gagea lutea* is a typical species of the species-rich sect. *Gagea* and inhabits deciduous woodlands and meadows of the humid temperate zone. *Gagea graeca* (sect. *Antericoides*) and *G. bulbifera* (sect. *Bulbiferae*) occur in arid habitats (Mediterranean shrub lands and Middle/Central Asian steppes, respectively). The latter two species assume different positions in phylogenies of the genus (Peterson et al. 2008), but share nevertheless several key traits that are different from *G. lutea*. First, both species from arid areas have high fecundity (as indicated by the number of seeds per produced per capsule, Table 2). Second, the threshold for flower formation is lower than for the formation of bulbils. Third, only a single basal bulbil is formed. Fourth, an ontogenetic switch between bulbil and seed production is absent: bulbils and seeds can be produced simultaneously (see discussion below). Fifth, the genetic diversity, calculated as the proportion of singleton genotypes, is rather high due to dominating reproduction via seeds.

Another difference between species from humid and arid regions regards seed morphology: Together with the species of the section *Bulbiferae*, the two species of sect. *Anthericoide*s share one important character (which may be ancestral for the genus, since it is occurring as well in other *Liliaceae* genera, Peruzzi 2016): the platyspermous (flattened) seeds. Several other sections (*Platyspermum*, *Plecostigma*, *Stipitatae*) of the genus *Gagea* are platyspermous, and these assume different positions in phylogenetic trees (Peterson et al. 2008). Many of these species are adapted to arid conditions and inhabit steppes of the Irano-Turanian and Saharo-Arab regions. One possible explanation would be to assume an adaptation to wind dispersal of seeds (Peterson et al. 2008). The comparison with *G. lutea* (Table 2), a species from humid environments with terete seeds, shows that the low seed weight seems to allow platyspermous species to produce more seeds. This should be an important precondition for an ephemerous life style. Seeds of the humid-zone species are about 20 times heavier than these of the two arid-zone species. Not surprisingly, *Gagea lutea* produced on average 41 seeds per flowering plant, the average for the studied populations of *G. graeca* is 68 (but it should be noted that ca. one-third of the sampled plants failed to set seed); and for *G. bulbifera* 119 seeds per plant were counted (Table 2). The difference between the species from humid and arid environments becomes obvious if seed production is compared with the average size of replacement bulbs: if bulb volume is estimated as that of a sphere from the figures for diameter (Table 2), *G. lutea* produces 0.1 seeds/mm^3^ bulb volume, but numbers for *G. graeca* and *G. bulbifera* are 4.8 and 13.7, respectively. Figures for bulbil production per mm^3^ replacement bulb volume are comparable (0.13 vs. 0.07 and 0.11 bulbils/mm^3^ bulb volume). Due to the switch for *G. lutea* the latter figure was calculated from the average dimeter of bulbil-producing, non-flowering plants (5.00 ± 1.51 mm). This indicates a clear shift in reproductive output from bulbils (*G. lutea*) towards seeds (*G. bulbifera*, *G. graeca*).

*Gagea lutea* differs from the two arid-zone species by another important character: a switch in ontogenesis between bulbil production and seed production (Schnittler et al. 2009). If plants gain resources (indicated by larger replacement bulbs), they produce first only bulbils, not flowers. Above a certain threshold in parent bulb size, only flowers, no bulbils, are developed. In such species, the resource threshold (expressed as diameter of the parent bulb) for bulbil production is always lower than that for flower/seed development (Schnittler et al. 2013). Species lacking a switch start to develop a single to several bulbils as soon as the replacement bulb has reached a certain diameter and then continue to form them indefinitely throughout the life of the plant. In such species, the threshold for flower/seed development may be lower than that for bulbil development, as it is the case in *G. bulbifera* and *G. graeca* (Table 2). From this reason, a direct comparison in terms of resources allocated to bulbils *vs.* seeds is impossible due to the switch in *G. lutea*: plants of this species never invest simultaneously into seeds and bulbils.

The results of AFLP genotyping reflect the consequences of a) seed and bulbil production and b) absence/presence of a switch for genetic diversity in a species. The AFLP data for *G. graeca* showed a small proportion of clonal plants (9%, Fig. 7), with only five pairs of clonal plants found in the first of the two transects. This is comparable with results from *G. bulbifera* (0% clonal plants, Beisenova et al. 2015) but not with the two remaining species of the genus with known data (*G. lutea*, 62%, Pfeiffer et al. 2011; *G. spathacea*, 100%, Pfeiffer et al. 2012). Only flowering plants of *G. graeca* were used for the AFLP analysis, which all can be expected to form one bulbil/year. In fact, in the neighborhood of a plant (the second member of a pair) we found in 50 cases a non-clonal plant (likely to be germinated from seeds, but we cannot rule out the possibility that they originate from the bulbil of a third plant which was not sampled), in five cases a clonal plant (derived from a bulbil of the first plant). If we compare these relationships (bulb to seed production 1:68 = 0.014; clonal to non-clonal descendants 5:50 = 0.100), we obtain roughly a sevenfold higher establishment rate of bulbils compared with seeds. The two structures are different in size: bulbils are nearly spherical and on average 0.48 mm in diam., equaling a volume of 0.46 mm^3^; seeds are flattened and approximately 0.1 × 0.15 × 1.3 mm, equaling a volume (if estimated as that of an ellipsoid) of 0.08 mm^3^. These differences should influence the amount of nutrients stored as well as the desiccation tolerance and explain why bulbils are likely to have a higher chance of establishment.

These considerations suggest that it is not the failure of bulbils to recruit new plants, but the comparatively high seed production of *Gagea graeca* that leads to a high genetic diversity of the populations. Seed production should nevertheless face a limit in arid climates: the more and the earlier in ontogenesis resources will be invested in seeds, the lesser resources remain for the replacement bulb which guarantees survival of the plant. But the relation volume to surface which we must assume to be an important parameter for desiccation tolerance, decreases with decreasing diameter of the bulb. In contrast to *G. bulbifera*, where bulbs can hold effectively water via sclerophyllous roots (see Levichev 1999), the bulbs of *G. graeca* lack this adaptation and may thus face a high risk of desiccation during the long Mediterranean summer. This limits the proportion of resources invested into seeds, since plants with large bulbs can be expected to have a higher chance of survival. Indeed, the average size of the bulbils is higher in *G. graeca* in comparison to *G. bulbifera* (3 vs. 2.5 mm), and so is the proportion of nitrogen invested into the replacement bulb (62% vs. 36%, Table 2). However, figures for relative investment into plant parts cannot be compared directly from two reasons: first, the measurements for *G. lutea* were obtained at the peak of flowering season, these for *G. bulbifera* near seed maturation, whereas the measured plants of *G. graeca* were already completely dried at the time of measurement. Therefore, storage processes may have been not yet completed in the first two species; which should lead to an underestimation of the relative investment into dormant parts (bulbs, bulbils, seeds) but overestimation for vegetative parts in the first two species. Second, *G. lutea* shows a switch – bulbils and seeds are never found at one plant, which does not allow a direct comparison.

Growth patterns in *Gagea* seem to be rather stable for a species (Levichev 2013). With a quantitative analysis of the morphology for a sampled population, thresholds for bulbil formation and flowering can be defined (Schnittler et al. 2009). For all hitherto investigated species from humid climates, thresholds for bulbil formation are lower than these for flower formation (Schnittler et al. 2013); the exception are the two species from arid climates, *Gagea bulbifera* and *G. graeca* (Table 2). Such shifts towards earlier generative reproductions may be a general trend for perennial herbs in arid habitats. Franks and Weis (2015) observed in a 5-year drought experiment with *Brassica rapa* that drought selected for plants that flowered at a smaller size and earlier ontogenetic stage. These changes seem to correspond with a lower level of neoteny and a much smaller genome in the arid-zone species (see discussion in Peruzzi et al. 2009). For *Gagea lutea*, direct measurements resulted in DNA contents (1C) of 19.75 pg (Greilhuber et al. 2000) and 21.35 pg (Zonneveld et al. 2015). Respective values for the species from arid environments, inferred from chromosome total haploid length are 4.00–6.96 (mean 5.48) pg for the species of sect. *Antericoides* and 8.65 pg for *G. bulbifera* (Peruzzi 2012a).

In contrast to the differences mentioned above, the trend towards andromonecy (weak plants produce male flowers only, and even in larger plants the last flower is often male) seems to be a general tendency in the genus. For *Gagea lutea*, this was reported by Nishikawa (1998), and male flowers were reported as well in the two species from arid zones. In the plants of *G. graeca* investigated for morphology we counted 95 flowering plants with one flower only, in 57 of these the flower was apparently hermaphroditic (mean bulb diam. 2.51 mm), in 38 it was apparently male (mean bulb diam. 2.31 mm). This is indirectly confirmed by the high proportion of weak sterile plants (30%) in the cohort sampled for seed set. An androdioecious breeding system was reported for several species in the genera *Gagea*, especially the species formerly assigned to *Lloydia* (Patterson and Givnish 2002; Manicacci and Despres 2001; Peruzzi et al. 2008). This fits into the pattern of frequent occurrence of female-sterile breeding systems observed in *Liliaceae* and was suggested to result from size-dependent sex allocation (Peruzzi 2012b). In addition to the finding that often the smallest plants of a cohort are male (*Lilium apertum*, Zhang et al. 2014; *Fritillaria montana*, Peruzzi et al. 2012), in *Gagea* spp. usually the last flowers, when the replacement bulb may run out of resources, are male. We did not investigate the pollination system for *G. graeca* but assume facultative autogamy, which was found as well in *G. lutea* (Pfeiffer et al. 2013). For *G. graeca*, Peruzzi et al. (2008) obtained viable seeds from isolated plants in cultivation.

In accordance with the general habit of the plants, our data suggest that the two species from arid environments (*Gagea graeca* and *G. bulbifera*), although belonging to divergent clades in phylogenetic trees, are more similar in life history than to *G. lutea* from a humid climate (Table 2). Not directly accessible is plant age, where the most remarkable differences may be expected. Since in *Gagea* the plants replace all structures of the vegetation body completely every year, and only the replacement bulb survives dormancy periods, only a mark-revisit approach over several years would provide reliable age data. In the field we found for *G. graeca* only a low proportion (28%) of non-flowering plants. Looking for the distribution of plants over size classes of 0.25 bulb diameter, the highest number of plants (56) was found for 2.50–2.75 mm diam. We therefore assume that *G. graeca* is a short-lived perennial and may flower and produce seeds already in the second year of its life (see also Peruzzi et al. 2008).

## Conclusions

Adaptations to arid environments in the genus *Gagea* (*Liliaceae*) are characterized by (i) a predominance of sexual reproduction and (ii) a lower reproductive age (lower threshold for flower development). This is connected with a reduction in size (especially bulb diameter). In contrast to most species of the genus, *Gagea graeca* seems to be a short-lived perennial, possessing several traits that are rather typical for annual plants.
